# Efficient generation of *GGTA1*-deficient pigs by electroporation of the CRISPR/Cas9 system into in vitro*-*fertilized zygotes

**DOI:** 10.1186/s12896-020-00638-7

**Published:** 2020-08-18

**Authors:** Fuminori Tanihara, Maki Hirata, Nhien Thi Nguyen, Osamu Sawamoto, Takeshi Kikuchi, Masako Doi, Takeshige Otoi

**Affiliations:** 1 Laboratory of Animal Reproduction, Faculty of Bioscience and Bioindustry, Tokushima University, 2272-1 Ishii, Myozai-gun, Tokushima, 779-3233 Japan; 2 Research and Development Center, Otsuka Pharmaceutical Factory, Inc., 115 Muya-cho, Naruto, Tokushima, 772-8601 Japan

**Keywords:** GGTA1, CRISPR/Cas9, Electroporation, In vitro fertilization, Pig

## Abstract

**Background:**

Xenoantigens are a major source of concern with regard to the success of interspecific xenografts. *GGTA1* encodes α1,3-galactosyltransferase, which is essential for the biosynthesis of galactosyl-alpha 1,3-galactose, the major xenoantigen causing hyperacute rejection. *GGTA1*-modified pigs, therefore, are promising donors for pig-to-human xenotransplantation. In this study, we developed a method for the introduction of the CRISPR/Cas9 system into in vitro-fertilized porcine zygotes via electroporation to generate *GGTA1*-modified pigs.

**Results:**

We designed five guide RNAs (gRNAs) targeting distinct sites in *GGTA1.* After the introduction of the Cas9 protein with each gRNA via electroporation, the gene editing efficiency in blastocysts developed from zygotes was evaluated. The gRNA with the highest gene editing efficiency was used to generate *GGTA1*-edited pigs. Six piglets were delivered from two recipient gilts after the transfer of electroporated zygotes with the Cas9/gRNA complex. Deep sequencing analysis revealed that five out of six piglets carried a biallelic mutation in the targeted region of *GGTA1,* with no off-target events. Furthermore, staining with isolectin B4 confirmed deficient *GGTA1* function in *GGTA1* biallelic mutant piglets.

**Conclusions:**

We established *GGTA1*-modified pigs with high efficiency by introducing a CRISPR/Cas9 system into zygotes via electroporation. Multiple gene modifications, including knock-ins of human genes, in porcine zygotes via electroporation may further improve the application of the technique in pig-to-human xenotransplantation.

## Background

Gene-modified pigs are ideal experimental animal models of human disease and donors for pig-to-human xenotransplantation [1–3]. As the life expectancy of humans increases, the incidences of chronic diseases and end-stage organ failure as well as demand for organ transplantation increase [2, 3]. In the context of a shortage in human organs for transplantation, pigs have gained importance as an alternative source. However, xenoantigens are a major source of concern for interspecific xenografts. Antibody–xenoantigen complexes lead to complement activation and immediate hyperacute rejection [4]. The major xenoantigen expressed in porcine tissues is the galactosyl-alpha 1,3-galactose (Galα(1,3)Gal) epitope, which is expressed on the surface of porcine endothelial cells and causes hyperacute rejection [3, 5].

α1,3-Galactosyltransferase, encoded by the glycoprotein galactosyltransferase alpha 1,3 (*GGTA1*), is essential for the biosynthesis of Galα(1,3)Gal [5]. The establishment of *GGTA1*-deficient pigs lacking the functional Galα(1,3)Gal epitope is a key step in controlling xenograft rejection. Since its establishment, somatic cell nuclear transfer (SCNT) has been the primary method for the generation of genetically modified pigs [6, 7]. Recently, gene editors such as zinc finger nuclease [8], transcription activator-like effector nuclease [9], and the clustered regularly interspaced short palindromic repeat (CRISPR)/CRISPR-associated (CRISPR/Cas) system [10–12], have improved gene modification activities markedly, including site-specific modification and gene knock-ins and knockouts. Gene editors have also enabled gene modification in porcine zygotes via direct injection into the cytoplasm [7]. However, micromanipulator systems for microinjections of gene editors or for nuclear transfer of donor cells in SCNT require sophisticated techniques, which limits the widespread generation of gene-modified pigs [6].

We recently developed the gene editing by electroporation of Cas9 protein (GEEP) method [13] in which the CRISPR/Cas9 system (Cas9 protein and guide RNA (gRNA)) is introduced into porcine zygotes by electroporation to disrupt a target gene. We also demonstrated that Cas9 mRNA electroporation into porcine zygotes resulted in a low gene-editing efficiency compared with Cas9 protein electroporation [13]. The electroporation procedure does not require sophisticated micromanipulation techniques. However, the efficiency of gene editing by GEEP and characteristics of piglets derived from electroporated zygotes have not been evaluated well, unlike in SCNT, in which the generation of genetically engineered pigs has been evaluated comprehensively [14]. To confirm the validity and efficacy of the approach, studies on the generation of genetically modified pigs from electroporated zygotes targeting various genes are required. *GGTA1* knockout pigs have been generated by SCNT [15–18], handmade cloning [19], and CRISPR/Cas9 microinjection into zygotes [20]; however, *GGTA1*-modified pigs have not been established by the electroporation of the CRISPR/Cas9 system into zygotes.

In this study, we generated *GGTA1*-edited pigs using the GEEP method to establish a resource to facilitate pig-to-human xenotransplantation studies and to confirm the efficiency of the method in the establishment of lines of genetically modified pigs.

## Results

### Experiment 1: comparison of the gene editing efficiency using different gRNAs

**Fig. 1:**
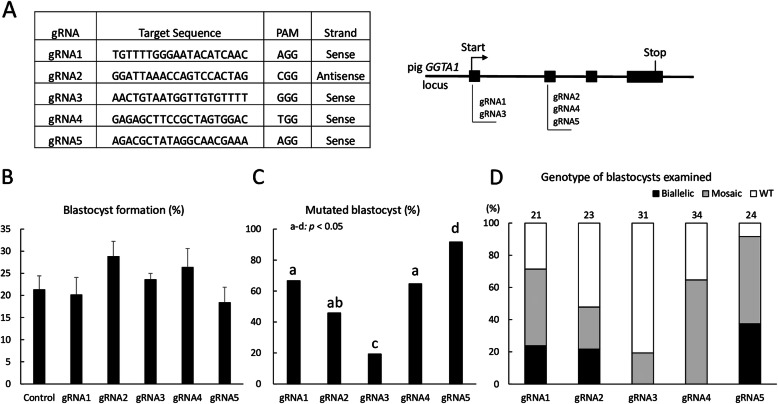
Confirmation of the gRNA gene-targeting efficiency. **a:** gRNA sequences targeting the *GGTA1* gene and genomic structure of the *GGTA1* locus. **b:** Blastocyst formation rates of the electroporated zygotes. For each treatment group, four replicates with 199–243 oocytes per treatment were analyzed. Values of means ± SEM are shown. **c:** Percentage of blastocysts carrying mutations in the *GGTA1* target region after zygote electroporation with the Cas9 protein and each gRNA targeting *GGTA1*. The percentage of mutant blastocysts was defined as the ratio of mutant blastocysts to the total blastocysts. Percentages of mutant blastocysts was analyzed by chi-squared tests. ^a–d^Values with different superscripts differ significantly (*p* < 0.05) and labels containing the same letter mean no significant difference. **d:** Genotypes of blastocysts determined by TIDE. WT, wild-type; Biallelic, biallelic mutant; Mosaic, mosaic mutant. Numbers above the bars indicate the total number of blastocysts examined

Five types of gRNA were designed (gRNA1–5) to target *GGTA1* (Fig. 1a). Each gRNA with the Cas9 protein was introduced into in vitro-fertilized zygotes by electroporation (five 1-ms square pulses at 25 V) of 100 ng/μl gRNA and 100 ng/μl Cas9 protein. The electroporation conditions have been evaluated in our previous study [21]. Thereafter, the blastocyst formation rate from electroporated embryos with introduced gRNA and the genotypes of obtained blastocysts were analyzed to evaluate their ability to develop to the blastocyst stage and the genome editing efficiency of each gRNA. No significant differences in blastocyst formation rates were observed among the experimental groups (Fig. 1b). The genotypes of blastocysts were determined by sanger sequencing and subsequent analysis using the TIDE (tracking of indels by decomposition) bioinformatics package [22]. In the present study, blastocysts carrying more than one type of mutation and the wild-type (WT) sequence were defined as mosaics. The proportion of mutant blastocysts harboring mosaic and biallelic mutants after the introduction of gRNA5 was significantly higher than the proportions after the introduction of other gRNAs (*p* < 0.05) (Fig. 1c). Using the Cas9/gRNA5 complex, 37.5% of blastocysts carried biallelic mutations (Fig. 1d). Although mosaicism complicates phenotypic analysis, non-mosaic *GGTA1*-deficient pigs can be generated by the subsequent breeding of F0 pigs carrying mosaic mutations. Therefore, we selected gRNA5 for use in the generation of *GGTA1*-edited pigs.

### Experiment 2: generation and analysis of GGTA1-edited piglets

**Fig. 2:**
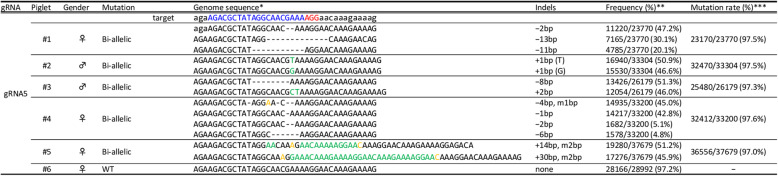
Deep sequence analysis of the *GGTA1* target region in delivered piglets. *Nucleotides in blue and red represent the target sequences and PAM sequences of each gRNA, respectively. Nucleotides in green and yellow represent inserted and modified sequences, respectively. **The frequency was defined as the ratio of the number of amplicons to the total read number. ***The mutation rate was defined as the ratio of the total number of mutant amplicons to the total read number. WT, wild-type; ♂, male; ♀, female

**Fig. 3:**
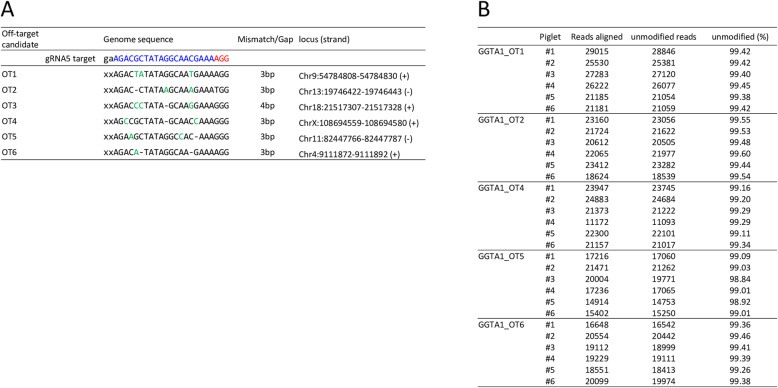
Off-target analysis of the delivered piglets via deep sequencing. **a:** Genome sequences and positions of possible off-target sites. Nucleotides in blue and red represent the target sequences and the PAM sequences of gRNA5, respectively. Nucleotides in green represent mismatches with the gRNA5 sequence. **b**: Frequency of the WT sequence at possible off-target sites

Cas9 protein and gRNA5 were introduced into approximately 400 zygotes by electroporation. Then, these zygotes were transferred into the oviducts of two recipient gilts (approximately 200 zygotes/gilts). Both recipient gilts became pregnant and gave birth to a total of six piglets. A deep sequencing analysis of DNA samples derived from ear biopsy samples of the delivered piglets was performed to evaluate gene editing in the target gene and off-target events. Deep sequencing of the *GGTA1* genomic regions flanking the target sites revealed that five out of the six piglets carried mutations in *GGTA1* (Fig. 2). None of the five piglets (#1, #2, #3, #4, and #5) had WT sequences; therefore, they were considered biallelic mutants. For an off-target analysis, we searched the whole genome sequence of the pig [UCSC (University of California, Santa Cruz) Genome Browser SGSC Sscrofa10.2/susScr3 assembly] for potential off-target sites and found six sites for gRNA5 with less than four mismatches/gaps (Fig. 3a). In a deep-sequencing analysis, we did not detect mutations at off-target sites in more than 99% of the amplicons (Fig. 3b). The remaining 1% was composed of a small number of amplicons (< 0.1%) carrying different sequences.

**Fig. 4:**
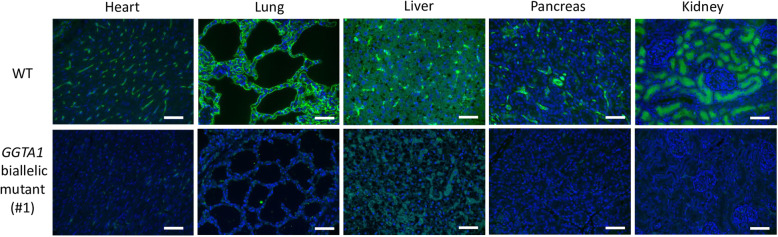
Immunohistochemical assessment of wild-type and *GGTA1* biallelic mutant piglets. The heart, lung, liver, pancreas, and kidney tissues derived from wild-type (WT) and *GGTA1* biallelic mutant piglets (#1) were immunohistochemically stained for αGal (green) and counterstained with DAPI (blue). The scale bar in each panel represents 50 μm

**Fig. 5:**
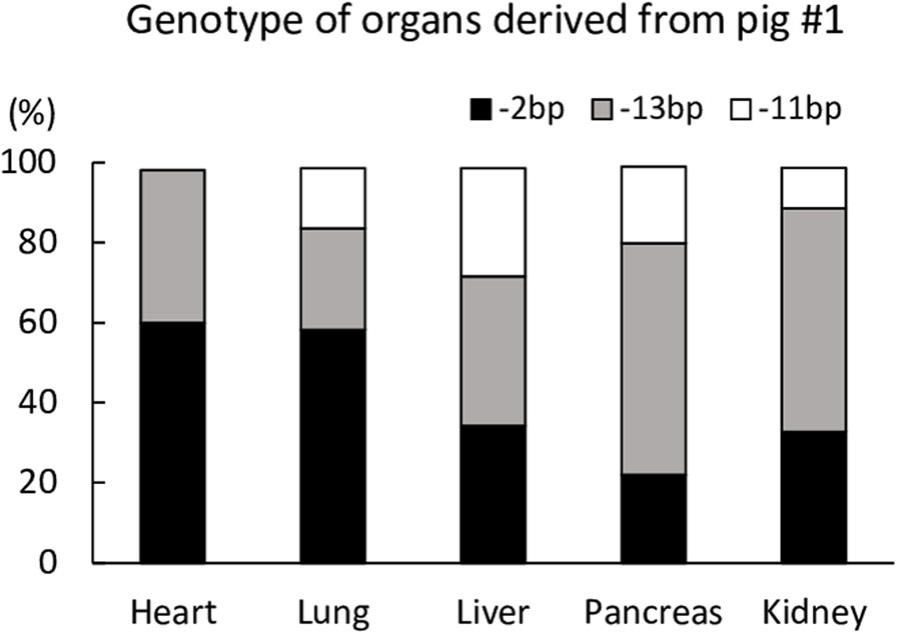
Genotype of major organs derived from piglets #1 analyzed by deep sequencing. Frequency of introduced mutations in selected organs

**Fig. 6:**
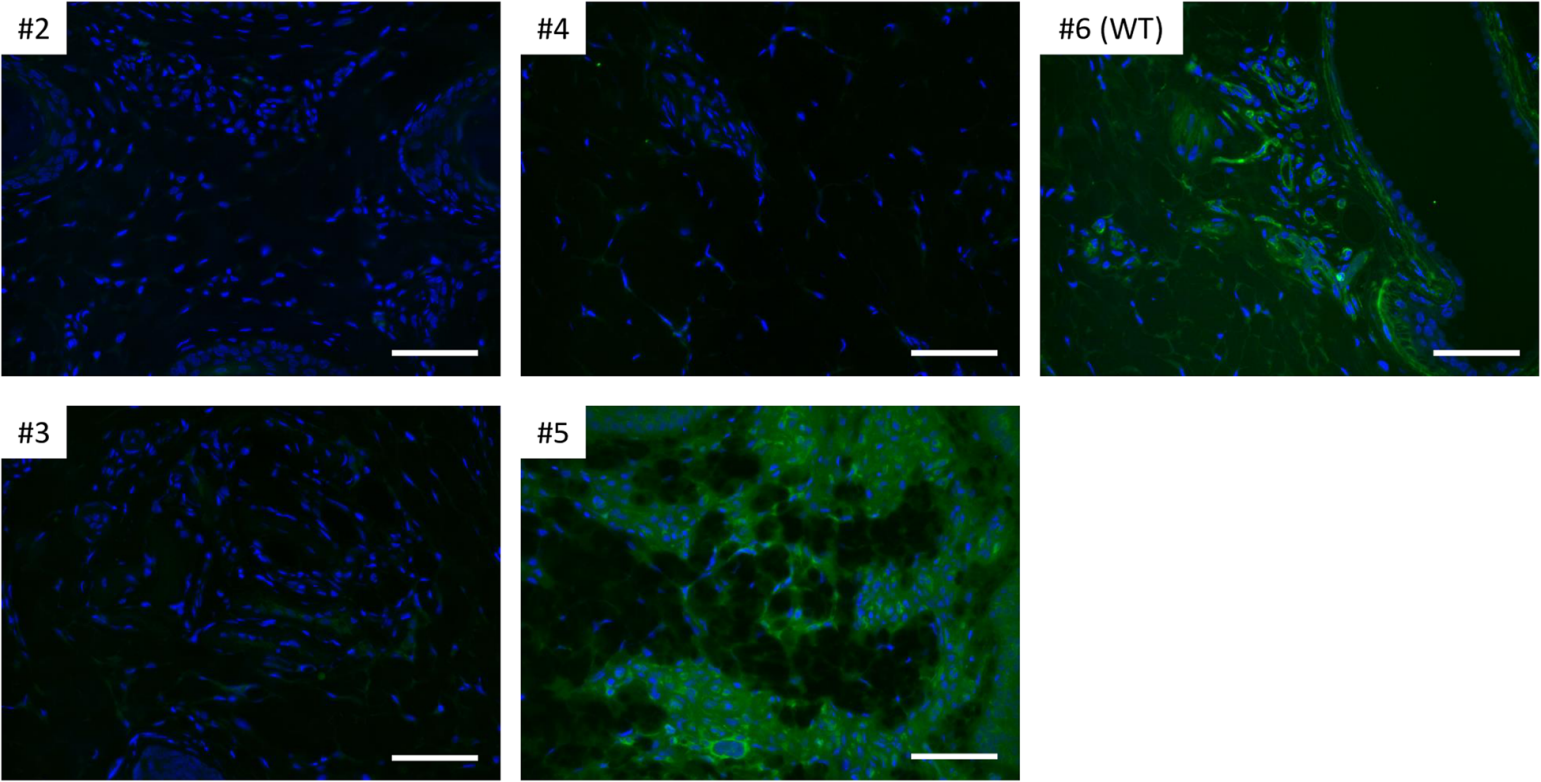
Comparison of the expression of αGal epitope in *GGTA1* mutant piglets with various genotypes by immunohistochemical assessment. The ear biopsy derived from wild-type (WT) and *GGTA1* biallelic mutant piglets (#2, #3, #4, and #5) were immunohistochemically stained for αGal (green) and counterstained with DAPI (blue). The scale bar in each panel represents 50 μm

The expression levels of the Galα(1,3)Gal epitope in heart, lung, liver, pancreas, and kidney tissues were assessed by staining using isolectin B4. The tissues derived from a *GGTA1* biallelic mutant piglet (#1) and its WT littermate (#6) were stained with Alexa 488-labeled isolectin B4 to analyze Galα(1,3)Gal epitope expression. A histological analysis indicated a deficiency in GGTA1 in the *GGTA1* biallelic mutant piglet (Fig. 4). The deep sequencing analysis of the genomic DNA derived from the heart, lung, liver, pancreas, and kidney of piglet #1 confirmed that these organs harbored the same type of mutations observed in the ear biopsy analyses; however, the frequency of these mutations varied with the organs (Fig. 5). Galα(1,3)Gal epitope expression was also assessed in ear biopsy samples from the other piglets (#2, #3, #4 and #5) and compared with that from a WT littermate (#6) (Fig. 6). Downregulation of Galα(1,3)Gal expression was observed in piglets #2, #3, and #4. The expression of Galα(1,3)Gal in Piglet #5 carrying in-frame mutation was similar to that in the WT.

**Fig. 7:**
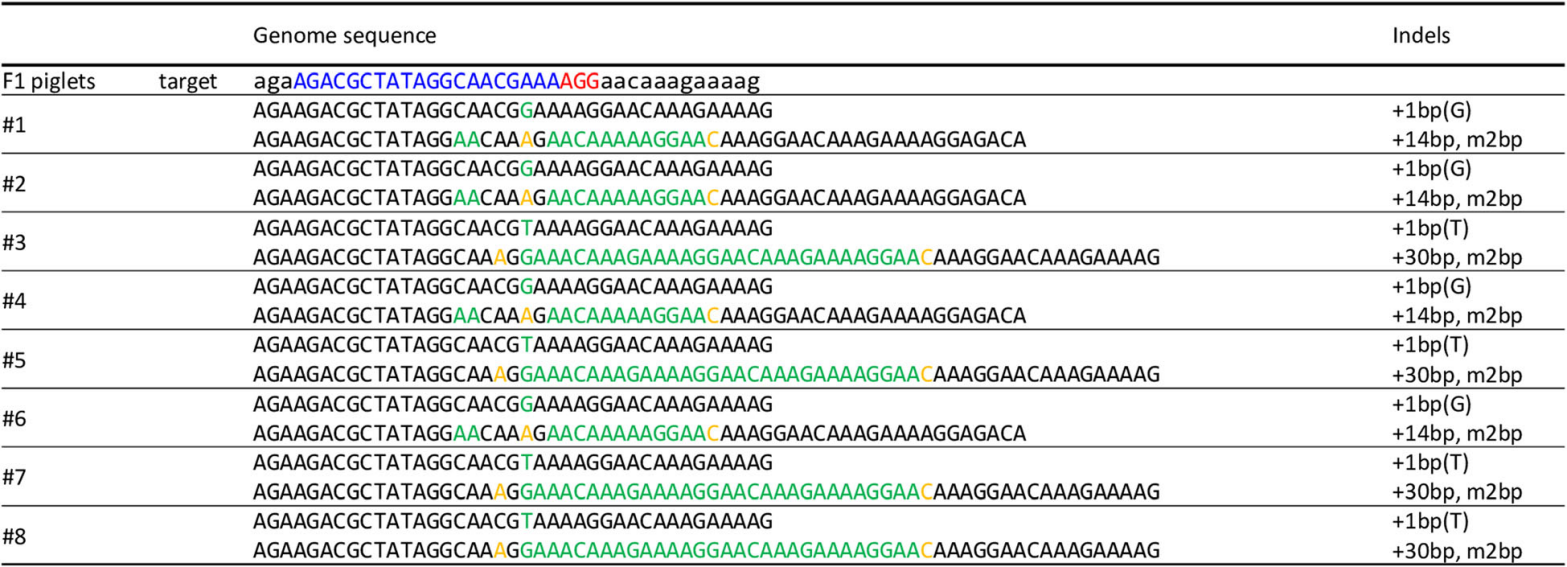
Analysis of the genome sequences of F1 piglets. The alignment of sequences from F1 piglets is shown. The nucleotides in blue and red represent the target sequences and the PAM sequences of gRNA5, respectively. The nucleotides in green represent the inserted sequences and those in yellow represent the modified sequences

Finally, we investigated whether the mutation detected in the F0 pig was inherited by the subsequent generation. We generated the F1 generation offspring by mating pig #2 (male) with #5 (female). Eight F1 piglets were delivered, and the piglets harbored mutations detected in the ear biopsy samples from pigs #2 and #5 (Fig. 7).

## Discussion

In the present study, we successfully generated *GGTA1* biallelic mutant pigs using the GEEP method based on the CRISPR/Cas9 system with high efficiency and no off-target events. The assessment of Galα(1,3)Gal epitope expression in ear biopsy samples and organ samples indicated that *GGTA1* biallelic mutant pigs without in-frame mutations exhibit the successful downregulation of the Galα(1,3)Gal epitope. Conversely, high frequency in-frame mutation (45.9%) seemed to result in the expression of Galα(1,3)Gal in one biallelic mutant piglet (#5).

Non-specific cleavage of off-target sequences are a major concern with respect to the practical implementation of the CRISPR/Cas9 system [23, 24]. In a deep sequencing analysis, more than 99% of the amplicons of potential off-target sites were made up of the WT sequence. The remaining 1% was composed of a small number of amplicons (< 0.1%) carrying different sequences. These were also detected when we analyzed a WT piglet by deep sequencing [13, 25], indicating that they may have been introduced by polymerase chain reaction (PCR) errors or sequencing errors. In addition, we introduced the CRISPR/Cas9 system into 1-cell-stage zygotes, indicating that off-target mutation carrying less than 0.1% of the sequences in individual offspring is difficult to achieve. Therefore, we concluded that no off-target events were detected in *GGTA1* mutant pigs generated in the present study. Varying degrees of off-target effects by CRISPR/Cas9 have been observed in cells [26, 27], and whole-genome sequencing analyses have identified off-target events in founder gene-modified mice generated using the CRISPR/Cas9 system [28]. Another study reported that off-target events are rare in gene-modified nonhuman primates generated using the CRISPR/Cas9 system [29]. In our previous study of the generation of *Myostatin* mutant pigs, no off-target events were observed [13]. Recently, mutant Cas9 nucleases with high specificity have been designed [30–32]. Such improvements could reduce off-target events, which should be carefully evaluated in gene-modified pigs supplied as founders.

In the present study, we achieved the efficient generation of *GGTA1* biallelic-mutant pigs using the GEEP method, in which the CRISPR/Cas9 system is introduced into zygotes by electroporation. The electroporation procedure does not require any sophisticated micromanipulation techniques, when compared with microinjection and SCNT medicated procedures, and the results of the present study demonstrated that the GEEP method is a promising approach for the generation of biallelic mutants with high efficiency. However, we have previously observed that the introduction of CRISPR/Cas9 into zygotes results in a high frequency of mosaicism in blastocysts and obtained piglets [13, 33, 34]. Based on an analysis of DNA derived from ear biopsy samples, piglet #1 showed three types of mutation; however, there could be differences in editing variants among the different organs. We performed a deep-sequencing analysis of the selected organs from piglet #1 for genotype confirmation. We observed that the piglet harbored mutations, as observed in the ear biopsy analysis, in its major organs at various frequencies. Our previous study demonstrated mosaicism of major organs with frequencies similar to those observed in the results of the ear biopsy analysis [35]. The frequency of genotype varies with organ in genetically modified F0 pigs, indicating that a careful observation of WT sequence/in-frame mutation in the genomic DNA is an essential step to apply these organs for transplantation.

The assessment of Galα(1,3)Gal expression in the major organs of piglet #1 demonstrated downregulation of Galα(1,3)Gal expression; however, the heart and liver tissues contained a Galα(1,3)Gal-positive region. Such residual amounts of Galα(1,3)Gal epitope reactivity, which may not be tissue specific, have also been observed in *GGTA1* knockout pigs [36] and cells [37]. Previous studies on *GGTA1* deficient pigs could not detect Galα(1,3)Gal expression on the vascular endothelium using the IB4 [16, 38]; therefore the α1,3-Galactosyltransferase has been believed to be the only enzyme that synthesizes Gal(1,3)Gal [39]. Milland et al. suggested that isoglobotrihexosylceramide 3 synthase (*iGb3S*) could also be responsible for Galα(1,3)Gal expression in *GGTA1* deficient mice and pigs as an alternative pathway [40]. However, Galα(1,3)Gal expression was observed in *GGTA1* and *iGb3s* double-knockout pigs [41]. Although Galα(1,3)Gal expression was successfully downregulated by *GGTA1* modification, another pathway could have facilitated the expression of Galα(1,3)Gal epitope in *GGTA1-*deficient pigs.

The direct introduction of the CRISPR/Cas9 system by zygote microinjection also suffers a risk of mosaicism [20, 42]. Mosaicism in the founder generation requires subsequent breeding for phenotypic analyses. In this study, inheritance analysis of *GGTA1*-modified F0 pigs demonstrated that the spectra of alleles present in the gametes was similar as the alleles observed in the ear biopsy; therefore, we will generate nonmosaic GGTA1 deficient pigs by mating using F0 pigs harboring the desired genotypes. However, subsequent breeding of F0 lines is cost- and time-intensive. Gene modifications by Cas9 protein/gRNA complexes remaining active during later developmental stages, following cleavage, can lead to mosaicism [43], which is presumed to be the reason for the more than two different genotypes in piglet #1 and #4 in the present study. This is expected to be reduced by the early delivery of the CRISPR/Cas9 system into zygotes. We previously evaluated the gene editing efficiency of the CRISPR/Cas9 system introduced before in vitro fertilization for the production of non-mosaic mutants [44]. Other studies have demonstrated that the microinjection of the CRISPR/Cas9 system into immature oocytes successfully reduces mosaicism [45, 46]. A combination of these strategies will reduce mosaicism by the one-step generation of gene-knockout founder pigs via microinjection and electroporation techniques.

*GGTA1* inactivation is an essential first step for controlling hyperacute rejection in pig-human xenotransplantation. However, other xenogeneic antigens, including beta-1,4-*N*-acetyl-galactosaminyltransferase 1 (B4GALNT) [47] and *N*-glycolylneuraminic acid synthesized by cytidine monophospho-*N*-acetylneuraminic acid hydroxylase (CMAH) [48, 49], also need to be eliminated to prolong organ survival. The triple knockout of porcine *GGTA1*, *CMAH,* and *B4GALNT2* has been achieved by SCNT and significantly reduces human IgG and IgM antibody binding to porcine peripheral blood monocytes, red blood cells [50–52], and the pericardium [53]. In addition, to prevent hyperacute rejection by controlling complement activation, the expression of human complement regulators in pigs has been studied [3, 54]. Other anti-inflammatory and anti-apoptosis-related genes in humans have also been expressed in pigs [55–57]. Xenograft rejection of these organs from genetically modified pigs to monkey and baboon models has been successfully delayed [58–60]. Multiple gene modifications, including knock-ins of human genes, will be essential for the maintenance of the function of xenotransplanted organs.

## Conclusion

Our method to establish *GGTA1*-modified pigs harboring mutations in their germ lines and without antigen-related hyperacute rejection is an efficient alternative to SCNT. The GEEP method does not require complex procedures. However, multiple gene-modified pigs generated using this method have not been established. To generate gene-modified pigs for pig-to-human xenotransplantation, further improvements aimed at the generation of multiple gene modifications, including knock-ins of human genes, in porcine zygotes, are required.

## Methods

### Animals

All animal care and experimental procedures, including the determination of experimental endpoints, were performed in accordance with the Guidelines for Animal Experiments of Tokushima University. All animals were housed and maintained in accordance with Institutional Animal Care and Use Committee guidelines. Two sexually mature Landrace gilts were obtained from the Tokushima Prefectural Livestock Research Institute (Tokushima, Japan) as recipients. Pigs were housed in a temperature-controlled room (25 ± 3 °C) under a 12-h light/12-h dark cycle with free access to water and were provided with commercial feed (JA Nishinihon Kumiai Shiryou, Hyogo, Japan). The health condition of all pigs was observed daily at feeding by the animal husbandry staff under the supervision of an attending veterinarian. To minimize animal suffering, all surgical procedures were performed under anesthesia by the intramuscular injection of 10 mg/kg ketamine (Ketalar, ketamine hydrochloride, Daiichi Sankyo Pharmaceutical, Tokyo, Japan) and continuous inhalation of 2 to 3% isoflurane (Mylan, Osaka, Japan) in the operation room. Euthanasia was performed by the intravenous injection of a potassium chloride solution (3 mmol/kg) under deep anesthesia by isoflurane according to the American Veterinary Medical Association Guidelines for the Euthanasia of Animals.

In this study, we obtained a total of six piglets. Five piglets (#1, #2, #3, #4, and #5) carried mutations in *GGTA1* gene, and one piglet was WT. For collecting tissue samples, piglets #1 and #6 were euthanized by the intravenous injection of a potassium chloride solution (3 mmol/kg) under deep anesthesia by isoflurane. Piglets #2, #3, #4, and #5 were maintained as a founder.

### Oocyte collection, in vitro maturation, and fertilization

Pig ovaries were obtained from prepubertal gilts (Landrace × Large White × Duroc breeds) at a local slaughterhouse and were transported in physiological saline within 1 h to the laboratory at 30 °C. Ovaries were washed three times with prewarmed physiological saline solution supplemented with 100 IU/ml penicillin G potassium (Meiji, Tokyo, Japan) and 0.1 mg/ml streptomycin sulfate (Meiji). Follicles with diameters of 3–6 mm on the ovarian surface were sliced on a sterilized dish using a surgical blade, and cumulus-oocyte complexes (COCs) were visualized and collected under a stereomicroscope. Approximately 50 COCs were cultured in 500 μl of maturation medium consisting of tissue culture medium 199 with Earle’s salts (TCM 199; Gibco/Invitrogen Co., Carlsbad, CA, USA) supplemented with 10% (v/v) porcine follicular fluid, 0.6 mM cysteine (Sigma-Aldrich, St. Louis, MO, USA), 50 μM β-mercaptoethanol (Wako Pure Chemical Industries Ltd., Osaka, Japan), 50 μM sodium pyruvate (Sigma-Aldrich), 2 mg/ml d-sorbitol (Wako Pure Chemical Industries Ltd.), 10 IU/ml equine chorionic gonadotropin (eCG; Kyoritu Seiyaku, Tokyo, Japan), 10 IU/ml human chorionic gonadotropin (hCG; Kyoritu Seiyaku), and 50 μg/ml gentamicin (Sigma-Aldrich), then covered with mineral oil (Sigma-Aldrich) for 22 h in 4-well dishes (Nunc A/S, Roskilde, Denmark). The COCs were transferred into a maturation medium without hormones for an additional 22 h. COCs were incubated at 39 °C in a humidified incubator containing 5% CO_2._

The matured oocytes were subjected to in vitro fertilization as described previously [61]. Briefly, frozen-thawed ejaculated spermatozoa were transferred into 5 ml of porcine fertilization medium (PFM; Research Institute for the Functional Peptides Co., Yamagata, Japan) and washed by centrifugation at 500×*g* for 5 min. The pelleted spermatozoa were resuspended in fertilization medium and adjusted to 1 × 10^6^ cells/ml. Then, approximately 50 oocytes were transferred to 500 μl of sperm-containing fertilization medium, covered with mineral oil in 4-well dishes, and co-incubated for 5 h at 39 °C in a humidified incubator containing 5% CO_2,_ 5% O_2_, and 90% N_2_. After co-incubation, the putative zygotes were denuded from the cumulus cells and the attached spermatozoa by mechanical pipetting, transferred to porcine zygote medium (PZM-5; Research Institute for the Functional Peptides Co.), and cultured for 7 h until electroporation.

### Electroporation

Electroporation was performed as described previously [13]. Briefly, an electrode (LF501PT1–20; BEX, Tokyo, Japan) was connected to a CUY21EDIT II electroporator (BEX) and was set under a stereoscopic microscope. The inseminated 50 zygotes were washed with Opti-MEM I solution (Gibco/Invitrogen, Carlsbad, CA, USA) and were placed in a line in the electrode gap in a chamber slide filled with 10 μl of Nuclease-Free Duplex Buffer (IDT; Integrated DNA Technologies, Coralville, IA, USA) containing 100 ng/μl gRNA (Alt-R CRISPR crRNAs and tracrRNA, chemically modified and length-optimized variants of the native guide RNAs purchased from IDT) targeting *GGTA1* and 100 ng/μl Cas9 protein (Guide-it Recombinant Cas9; Takara Bio, Shiga, Japan). According to manufacturer’s instruction, crRNA contains chemical modifications to protect it from degradation by cellular RNases, and tracrRNA contains chemical modifications conferring high nuclease resistance. gRNAs were designed using the CRISPRdirect webtool (https://crispr.dbcls.jp/) [62]. To minimize off-target effects, the 12 bases at the 3′ end of the designed gRNAs had no identical sequence matches to the pig genome other than the target region of *GGTA1,* as determined using the COSMID webtool (https://crispr.bme.gatech.edu/), which scores and ranks off-target candidate sequences based on the locations and numbers of base mismatches, deletions, and insertions, when compared to the gRNA sequence [63].

After electroporation (five 1-ms square pulses at 25 V), the zygotes were washed with PZM-5 and were cultured until embryo transfer (for 12 h) or for 3 days. The embryos that were cultured for 3 days were subsequently incubated in porcine blastocyst medium (PBM; Research Institute for the Functional Peptides Co.) for 4 days to evaluate their ability to develop to the blastocyst stage and for blastocyst genotyping. As a control, some of the inseminated zygotes were cultured with PZM-5 and PBM for 7 days without electroporation. Zygotes and embryos were incubated at 39 °C in a humidified incubator containing 5% CO_2_, 5% O_2_, and 90% N_2_.

### Analysis of the targeted gene after electroporation

Genomic DNA was isolated from blastocysts by boiling in a 50 mM NaOH solution. After neutralization, the genomic regions flanking the gRNA target sequences were PCR-amplified by the following specific primers: gRNA1 and gRNA3, 5′- AGTCAGGATGCTTCCCCTTT − 3′ (forward) and 5′- AAGCTGGTGACTTGGCTGAT − 3′ (reverse), gRNA2, gRNA4, and gRNA5, 5′- AAAAGGGGAGCACTGAACCT − 3′ (forward) and 5′- ATCCGGACCCTGTTTTAAGG − 3′ (reverse). The PCR products were extracted by agarose gel electrophoresis. The targeted genomic regions were directly sequenced. Sanger sequencing was performed using a BigDye Terminator Cycle Sequencing Kit ver. 3.1 (Thermo Fisher Scientific, Waltham, MA, USA) and the ABI 3500 Genetic Analyzer (Applied Biosystems, Foster City, CA, USA). The TIDE bioinformatics package [22] was used to determine the genotypes of blastocysts. Blastocysts were classified as having biallelic mutations (carrying no WT sequences), mosaics (carrying more than one type of mutation and the WT sequence), or WT (carrying only the WT sequence).

### Embryo transfer

Recipient gilts, the estrous cycles of which had been synchronized, were prepared for embryo transfer as described previously [64]. In brief, 0.2 mg of cloprostenol (Planate, MSD Animal Health, Tokyo, Japan) was given by intramuscular (i.m.) injection to pregnant gilts 35 to 53 days after the day of insemination. Subsequently, the second i.m. injection of 0.2 mg of cloprostenol and i.m. injection of 1000 IU of eCG (PMSA for Animal, ZENOAQ, Fukushima, Japan) were given to the gilts 24 h after the first injection of cloprostenol. At 72 h after the eCG i.m. injection, 1500 IU of hCG (Gestron 1500, Kyoritsu Seiyaku) was administered to the gilts. Approximately 72 h after the eCG i.m. injection, one- to two-cell stage embryos which were electroporated approximately 12 h before the embryo transfer were transferred into the oviducts of a recipient gilt under anesthesia. The gilts were placed in the supine position and the surgical area was disinfected with povidone-iodine (Meiji Seika Pharma, Tokyo, Japan). Generally, spontaneous ovulation rate, which varies across breeds, ranges from 10 to 23 [65, 66]. Viability and quality of in vitro-derived embryos are inferior to those of in vivo-derived embryos [67], and blastocyst formation rate was around 20% in the present study. Therefore, approximately 100 zygotes were transferred to each oviduct, resulting in the transfer of 200 zygotes (equivalent to approximately 40 blastocysts) per gilt under sterile conditions.

### Mutation analysis in piglets by deep sequencing

The ear biopsies were collected from piglets under anesthesia by continuous inhalation of 2 to 3% isoflurane. Genomic DNA was isolated from the ear biopsies by boiling in a 50 mM NaOH solution. After neutralization, the genomic regions that flanked the gRNA target sequences were amplified by two-step PCR by specific primers (S1 Table) and the Index PCR Primers following the manufacturer’s instructions (Illumina, Hayward, CA, USA). After gel purification, the amplicons were subjected to MiSeq sequencing using the MiSeq Reagent Kit v. 2 (250 cycles) (Illumina, San Diego, CA, USA). The mutation rates were defined as the ratio of the number of mutant amplicons to the total read number. A small number of amplicons carrying different sequences, that were also detected in WT samples were excluded as sequencing errors. Piglets that carried no WT sequences were classified as having biallelic mutations. Those carrying more than one type of mutation and the WT sequence were classified as mosaics. Piglets that carried only the WT sequence were classified as WT.

For the evaluation of genotype in major organs, genomic DNA isolated from the heart, lung, liver, pancreas, and kidney were amplified by two-step PCR and subjected to MiSeq sequencing as described above.

### Off-target effects determined by deep-sequencing

Off-target analysis was performed as described previously [13]. The COSMID webtool was used to determine the predicted off-target candidates. The genomic regions flanking potential off-target sites were amplified by two-step PCRs using specific primers (S2 Table) and the Index PCR Primers following the manufacturer’s instructions (Illumina), and subjected to a MiSeq sequencing analysis. Indel or substituted mutations were measured within a 5-bp window around the predicted Cas9 cleavage site in each off-target sites using CRISPResso [68] to minimize false-positive classification. A small number of amplicons carrying different sequences that were also detected in a WT sample were excluded as sequencing errors.

### Immunohistochemical assessment of piglets

A *GGTA1* biallelic mutant piglet and its WT littermate were euthanized by the intravenous injection of a potassium chloride solution (3 mmol/kg) under the intramuscular injection of 10 mg/kg ketamine and subsequent deep anesthesia by isoflurane. Tissues were dissected, fixed in a 4% paraformaldehyde neutral-buffered solution (Wako, Osaka, Japan), and manually embedded in paraffin. The paraffin-embedded sections were deparaffinized in xylene and rehydrated in decreasing concentrations of ethanol. Blocking treatment was performed by incubation with 0.1% bovine serum albumin in phosphate buffered saline supplemented with 1 mM CaCl_2_, 1 mM MgCl_2_, and 1 mM MnCl_2_ for 30 min at 25 °C. The sections were incubated overnight with 10 μg/ml isolectin B4-Alexa 488 (Thermo Fisher Scientific) at 4 °C and were subsequently fixed with 10% neutral formalin. The nuclei were counterstained with DAPI.

### Statistical analyses

Percentage data for embryos that developed to the blastocyst stage were subjected to arcsine transformation before analysis of variance (ANOVA). The transformed data were evaluated by ANOVA, followed by protected Fisher’s least significant difference tests. The analysis was performed using StatView software (Abacus Concepts, Berkeley, CA, USA). The percentages of mutated blastocysts were analyzed using chi-squared tests. Differences with a probability value (*p*) of 0.05 or less were considered statistically significant.

## Supplementary information

**Additional file 1 S1 Table.** Oligonucleotide sequences used for analysis of the introduced mutations in piglets by deep sequencing. **S2 Table.** Oligonucleotide sequences used for off-target analysis by deep-sequencing.

## Data Availability

The datasets used and/or analysed during the current study are available from the corresponding author on reasonable request.

## Abbreviations

ANOVA: ANalysis Of VAriance
B4GALNT: Beta-1,4-N-acetyl-GALactosaminylTransferase 1
Cas9: CRISPR-associated system 9
CMAH: Cytidine Monophospho - N - acetylneuraminic Acid Hydroxylase
COCs: Cumulus-Oocyte Complexes
CRISPR: Clustered Regularly Interspaced Short Palindromic Repeat
eCG: Equine Chorionic Gonadotropin
gRNA: Guide RNA
GEEP: Gene Editing by Electroporation of Cas9 Protein
GGTA1: Glycoprotein Galactosyl Transferase Alpha 1,3
hCG: Human Chorionic Gonadotropin
iGb3s: Isoglobotrihexosylceramide 3 synthase
PAM: Protospacer Adjacent Motif
PBM: Porcine Blastocyst Medium
PCR: Polymerase Chain Reaction
PFM: Porcine Fertilization Medium
PZM-5: Porcine zygote medium-5
SCNT: Somatic Cell Nuclear Transfer
TCM199: Tissue Culture Medium 199
TIDE: Tracking of Indels by DEcomposition
WT: Wild-type
