# Supplementary material for: Efficient generation of GGTA1-deficient pigs by electroporation of the CRISPR/Cas9 system into in vitro-fertilized zygotes
Source: BMC Biotechnol. 2020 Aug 18;20:40. doi: 10.1186/s12896-020-00638-7 (PMC7436961; doi:10.1186/s12896-020-00638-7)
Supplement: Supplementary file 1 — Additional file 1 S1 Table. Oligonucleotide sequences used for analysis of the introduced mutations in piglets by deep sequencing. S2 Table. Oligonucleotide sequences used for off-target analysis by deep-sequencing. [file 12896_2020_638_MOESM1_ESM.docx]

**S1 Table. Oligonucleotide sequences used for analysis of the introduced mutations in piglets by deep sequencing**

| Primer | | Common sequence | |  | Specific sequence |
| --- | --- | --- | --- | --- | --- |
| Forward | | ACACTCTTTCCCTACACGACGCTCTTCCGATCT | |  | CGTGTTCTCTGCCTTGGAAT |
| Reverse | | GTGACTGGAGTTCAGACGTGTGCTCTTCCGATCT | |  | ATCCGGACCCTGTTTTAAGG |
|  | |  |  |  |  |

**S2 Table. Oligonucleotide sequences used for off-target analysis by deep-sequencing**

| Primer | Off-target candidate | Common sequence |  | Specific sequence |
| --- | --- | --- | --- | --- |
| Forward | OT1 | ACACTCTTTCCCTACACGACGCTCTTCCGATCT |  | GGCAGTATTTTTCAACTCACAAG |
|  | OT2 |  |  | AAATCTTGCACACATACGGAAG |
|  | OT3 |  |  | CAAAGGCCTTGAGTCAGGAG |
|  | OT4 |  |  | CAGTGGCTACAGCTCCGATT |
|  | OT5 |  |  | GTGGGGCTGAAAGTGTGAGT |
|  | OT6 |  |  | GCTGGGTCTCTTGTCACACC |
| Reverse | OT1 | GTGACTGGAGTTCAGACGTGTGCTCTTCCGATCT |  | AGGCTTGTGAATCCTTCACTG |
|  | OT2 |  |  | GCTGACAGATATCAAAAGTCCAGT |
|  | OT3 |  |  | TGACCTCACCTGTGTTTCCA |
|  | OT4 |  |  | GGGATGCAGCAGAGTGTTTT |
|  | OT5 |  |  | TTTCCTACCTCACCTCAAGGAA |
|  | OT6 |  |  | CATGGCAGTTCCAAAAGAGG |
